# Drying of Functional Hydrogels: Development of a Workflow for Bioreactor-Integrated Freeze-Drying of Protein-Coated Alginate Microcarriers for iPS Cell-Based Screenings

**DOI:** 10.3390/gels11060439

**Published:** 2025-06-07

**Authors:** Johnn Majd Balsters, Alexander Bäumchen, Michael Roland, Stefan Diebels, Julia C. Neubauer, Michael M. Gepp, Heiko Zimmermann

**Affiliations:** 1 Fraunhofer Institute for Biomedical Engineering IBMT, 66280 Sulzbach, Germany; johnn-majd.balsters@ibmt.fraunhofer.de (J.M.B.); julia.neubauer@ibmt.fraunhofer.de (J.C.N.); michael.gepp@ibmt.fraunhofer.de (M.M.G.); 2 Fraunhofer Project Center for Stem Cell Process Engineering, 97070 Wuerzburg, Germany; 3 Molecular and Cellular Biotechnology/Nanotechnology, Saarland University, 66123 Saarbruecken, Germany; 4 Applied Mechanics, Saarland University, Campus, 66123 Saarbruecken, Germany; alexander.baeumchen@uni-saarland.de (A.B.); michael.roland@uni-saarland.de (M.R.); s.diebels@mx.uni-saarland.de (S.D.); 5 Faculty of Marine Science, Universidad Católica del Norte, Larrondo 1281, Coquimbo 1780000, Chile

**Keywords:** suspension bioreactor, drug discovery, pluripotent stem cells, freeze-drying, (UHV)-alginate, tissue engineering

## Abstract

Protein-coated ultra-high viscosity (UHV)-alginate hydrogels are essential to mimic the physiological in vivo environment of humans in several in vitro applications. This work presents an optimized bioreactor-integrated freeze-drying process for Matrigel^TM^-coated UHV-alginate microcarriers in the context of human induced pluripotent stem cell (hiPSC) expansion. The impact of freeze-drying on the UHV-alginate microcarriers using trehalose 100 mg/mL in 0.9% NaCl as a lyoprotective agent, as well as the stem cell response using hiPSCs, was analyzed using microscopy-based screenings. First observations of the process showed that the integrity of the cake was preserved in the samples with a maximum vapor exchanging rate. Following rehydration, the UHV-alginate microcarriers retained their original morphology. Upon the addition of Poloxamer 188, stickiness and bubble formation were reduced. The expansion of hiPSCs in a suspension bioreactor resulted in a 5–7-fold increase in total cell count, yielding at least 1.3 × 10^7^ cells with viability exceeding 80% after seven days of cultivation. In flow cytometry analysis, the pluripotency factors OCT3/4 and SSEA4 resulted in positive signals in over 98% of cells, while the differentiation factor SSEA1 was positive in fewer than 10% of cells. Supported by preceding in silico predictions of drying time, this study presents, for the first time, basic steps toward a “ready-to-use” bioreactor-integrated freeze-drying process for UHV-alginate microcarriers in the iPSC context.

## 1. Introduction

Biocompatible biomaterials have become indispensable in biomedical and tissue engineering applications. As one of the most important representatives of biomaterials, hydrogels are increasing in importance in human stem cell-based applications. These hydrogels, such as ultra-high viscosity (UHV)-alginates, are of great interest as they address the unmet need for physiological in vivo environments in in vitro applications [1]. Traditionally, cells, e.g., human induced pluripotent stem cells (hiPSC), are cultivated in planar polystyrene dishes. The cultivation on such plastic surfaces not only limits cell–cell and cell–matrix interactions but limits also the surface’s stiffness and scalability [2,3,4]. In particular, the scalability of the growth area is a crucial challenge, since almost all biomedical applications, e.g., cytotoxicity testing and drug screenings, require high cell quantities (>10^8^ cells per application) [5]. To provide high cell quantities, robust and scalable cell expansion processes are needed. A promising tool to facilitate cell expansion is the application of microcarriers in suspension bioreactors (SBRs). Microcarriers are spherically shaped and exhibit the highest surface-to-volume ratio [6,7]. Thereby, expansion processes and growth area can easily be scaled up by adding more microcarriers into the SBR without increasing the volume or the number of vessels. Most commercially available microcarriers are either made of stiff polystyrene (e.g., Corning Synthemax^®^, Cytodex^TM^ 1, SoloHill^®^) or are dextran based with undefined stiffness (Cytodex^TM^ 3) (Table 1).

Table 1 shows commercially available cultivation surfaces with different characteristics, clearly demonstrating a lag in “ready-to-use” soft microcarriers. Furthermore, the available microcarriers need further activation and process steps until usage inside a SBR for cell expansion. To overcome this limitation, the use of UHV-alginate microcarriers is promising and already established in several hiPSC applications [27]. In particular, UHV-alginate microcarriers were already successfully used for hiPSC expansion processes [27]. The UHV-alginates used in this study were extracted from brown algae and consist of two 1,4-linked monomers: β-D-mannuronic acid (M) and α-L-guluronic acid (G) [28,29,30]. The production of fresh microcarrier for, e.g., hiPSC expansion involves several complex and time-consuming steps. First, alginate sol is added dropwise into a cross-linking solution to create alginate (hydrogel) beads. After the production of alginate beads and cross-linking using Ba^2+^, the bioinert alginate beads must be functionalized according to Gepp et al. [31]. The UHV-alginate microcarriers are, e.g., protein-coated with Matrigel^®^ (MTG) or collagen I, which enables the cultivation of anchorage-dependent cells, such as stem cells. Afterwards, the modified microcarriers must be stored in an isotonic 0.9% NaCl solution at 4 °C. Since the protein-coated UHV-alginate microcarriers are stored in an aqueous solution, several negative side effects occur that influence their physicochemical characteristics, integrity, and functionality. During storage of an ionotropic hydrogel in aqueous solution, ions from the hydrogel and the aqueous environment exchange, which directly influences the hydrogel’s physicochemical properties [32,33]. This ion exchange was observed by Saitoh et al. who describes for calcium-alginate hydrogels a release of Ca^2+^ into the solution and a movement of Na^+^ from the solution into the hydrogel. This movement results in changes in physicochemical characteristics that influence the cell behavior on the UHV-alginate microcarriers. Another important effect described in the literature is the degradation of alginate hydrogels in aqueous solution (e.g., cell culture media). Depending on the molecular weight or chemical modification (e.g., oxidation), the mechanical properties are influenced, as described by Boontheekul et al. [34]. Furthermore, the storage ability of proteins in liquid solutions is limited, since denaturation of the protein layer during storage in liquid solution occurs [35,36,37]. The denaturation of proteins related to cell adhesion cause a loss in functionality; hence, hiPSCs cannot attach and spread on the hydrogel surface. In addition, UHV alginate and MTG are natural products, exhibiting batch-to-batch variation. Aisenbrey et al. describes differences in MTG stiffness, even significant local differences in stiffness within a single batch [38,39]. MTG is a semi-defined protein mixture, and its composition varies from batch to batch [40]. These properties cannot be influenced or are difficult to influence and lead, in consequence, to lack in reproducibility in cell expansion and cell–matrix interaction understanding. Additionally, UHV alginate can differ from batch to batch, e.g., in the G/M ratio, which, after further modifications, leads to different mechanical characteristics and rheological behavior [41,42]. To address these challenges and limitations, it is imperative to establish an alternative and improved storage method. The overarching objective is to mitigate both chemical and biological activity, as well as the negative impacts of batch-to-batch variability. Since the adverse effects primarily occur during storage in liquid solutions, implementing a drying method for long-term storage appears to be a sustainable solution. Among various drying techniques, freeze-drying is scalable and noted as the gentlest method for hydrogels [43]. Freeze-drying is a process operated in three steps [44]. In the current state-of-the-art approach, the sample is frozen until total solidification. After solidification, a vacuum is added to the system to remove frozen water by sublimation [45]. The last step includes an increase in temperature to remove bound water by desorption [46]. Using freeze-drying, one UHV-alginate batch and one MTG batch can be processed, modified, freeze-dried for long-term storage, and utilized after several months or years. Hereby, batch-to-batch differences are eliminated, and the interpretation of biological data is facilitated. Previously, freeze-drying has already been applied in several studies involving alginate-based or alginate-based hybrid hydrogels. In most applications, freeze-drying has been employed either to tailor the microstructure and induce porosity [47,48,49,50,51,52,53,54] or to investigate a preparation strategy for SEM analysis of alginate-based hydrogels [55]. Shapiro et al. employed freeze-drying to generate a porous alginate sponge, onto which fibroblasts were seeded [50]. They observed that the cells predominantly adhered and proliferated within the pores while maintaining their round morphology [50]. In addition, for freeze-dried alginate-based microcarriers, parameters such as structural stability under culture conditions, swelling behavior, and Young’s modulus were assessed [56]. While Chui et al. included mechanical characterization of the freeze-dried alginate microcarriers, a subsequent biological evaluation, in particular cell growth studies, was lacking. As a consequence, the current literature and state-of-the-art reveals a gap in scalable, “ready-to-use” alginate microcarriers in the context of freeze-drying strategies for stem cell-based applications. In this work, the previously described approach will be further enhanced for applications in stem cell technology, and an initial approach toward a “ready-to-use” freeze-dried microcarrier-based SBR process is presented. Protein-coated UHV-alginate microcarriers were freeze-dried directly in the SBR using trehalose as a lyoprotective agent. Freeze-drying directly in the SBR minimizes intermediate steps, thereby reducing the loss of functional cultivation surface. Following the freeze-drying process, functional tests were conducted by expanding hiPSCs on the reconstituted UHV-alginate microcarriers and analyzing cell count as well as the maintenance of their pluripotency status.

## 2. Results and Discussion

### 2.1. Simulation of a Modified Freeze-Drying Process for “Ready-to-Use” Alginate Microcarrier Hydrogels

**Figure 1:**
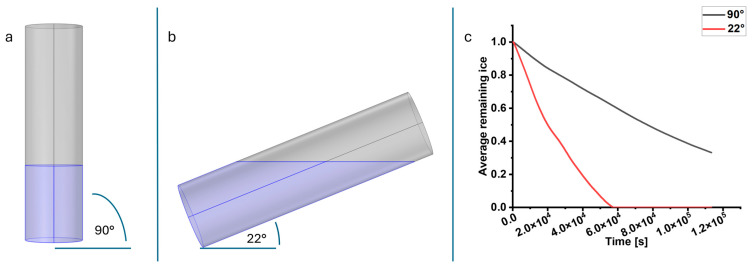
Simulation of two different setups with simplified SBR 90° upright to the shelf (**a**) and 22° tilted to the shelf (**b**). The simulation shows a significant faster water removal for the 22° oriented SBR (**c**). The average remaining ice for the 22° oriented SBR reaches zero after approximately 15.5 h.

For the implementation of bioreactor-integrated functional freeze-drying of MTG-coated UHV-alginate microcarriers, an initial in silico verification was setup to confirm the effectiveness of the designed process. The average normalized remaining ice content for both the upright (90° oriented) and the tilted (22° oriented) setup is shown in Figure 1.

The contents of the 22° oriented SBR dry significantly faster, reaching 0% ice content after approximately 15.5 h, whereas the 90° oriented SBR retains about 33% after the full 31.5-h duration. This is attributed to the increased surface area of the frozen solution exposed to the vacuum, leading to higher heat and mass transfer.

### 2.2. Implementation of a Freeze-Drying Process for “Ready-to-Use” Alginate Microcarrier

**Figure 2:**
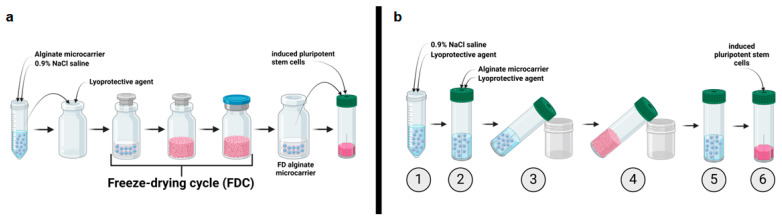
Freeze-drying strategies. (**a**) State-of-the-art freeze-drying cycle from loading to cell inoculation. The UHV-alginate microcarriers must be transferred from the initial tube into the lyo-vial along with the lyoprotective agent. The sample, together with the lyoprotective agent, is then transferred to the freeze-dryer where the freeze-drying cycle (FDC) can be initiated. After the cycle, the sample must be rehydrated and transferred to the vessel required for the experiment. (**b**) Freeze-drying cycle for ready-to-use microcarriers in a bioreactor. The UHV-alginate microcarriers are stored in a 0.9% isotonic NaCl solution (1) and are poured together with the lyoprotective agent formulation directly into the SBR that is intended for the cultivation process (2). The SBRs were positioned at an angle in the freeze-dryer to maximize the surface area and accelerate the process (3). Subsequently, the freeze-drying process is initiated until the samples were dried (4). Afterwards, the dried samples were rehydrated, and the solution was aspirated (5). In the final step (6), the iPSCs are inoculated onto the rehydrated UHV-alginate microcarriers. Figure created with BioRender.com.

In this study, a bioreactor-integrated freeze-drying process was implemented. Samples are typically freeze-dried in lyo-vials. This process involves transferring the samples from their original tubes into the lyo-vials and then into the required vessel after freeze-drying (Figure 2a). Reducing the transfer steps lead to a decrease of cultivation surface loss and facilitates handling during the process. Figure 2b schematically illustrates the concept of a bioreactor-integrated functional freeze-drying process. The protein-coated UHV-alginate microcarriers along with the lyoprotective agent were directly transferred into the SBR used for the cultivation process (see Figure 2b, steps 1 and 2).

This approach eliminated unnecessary transfer steps and reduced the loss of functional cultivation surface. The conventional freeze-drying process for protein-coated UHV-alginate microcarriers has several bottlenecks, with loss of growth surface occurring in the worst case scenario in three situations. The first situation is the loss of microcarriers that stick to the inner wall of the glass vial. Only with time-consuming mechanical and manual “pull-down” support can individual microcarriers be transferred into suspension for further utilization. The second bottleneck involves the microcarriers that stick to the bottom edge of the glass vial. Using time-consuming resuspending steps, single microcarrier can be collected with the pipette tip. The third bottleneck is the final transferring step from the lyo-vial into the bioreactor. During the transfer from the glass vial to SBR, microcarriers can be stuck inside the pipette tip, and their further utilization is almost impossible. During the freeze-drying cycle, the SBR was positioned at an angle to accelerate the freezing and drying by increasing the available surface of the solution. Tilting the SBR increases the efficiency of the process. After the cycle, the cakes were rehydrated inside the SBR. Then, the liquid was aspirated, and the hiPSCs were inoculated onto the reconstituted UHV-alginate microcarriers. As the freeze-drying process was conducted within the final vessel, initial steps toward a “ready-to-use” SBR for hiPSCs expansion process have been taken.

### 2.3. Analysis of Lyophilized Cake Appearance

**Figure 3:**
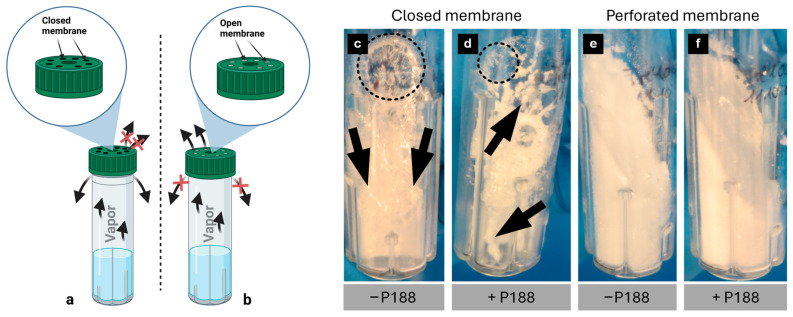
Schematic illustration of sublimation systems and cake appearances. Water vapor escaping possibilities during sublimation. (**a**) The lid was not totally closed to enable escaping possibilities between the lid and bioreactor. The membrane therefore was left closed. (**b**) The lid was closed properly to prevent the exit of the vapor on the interspace. Instead, the membrane was perforated to enable vapor exit through the lid. Figure created with BioRender.com. Different cake appearances depending on the water vapor escaping possibilities. When the membrane remains closed and vapor is exclusively escaping through the interspace, the lyo-cake reveals two morphological characteristics: collapse (dashed circle) and meltback (arrows) (**c**,**d**). When the exit is occurring through the perforated membrane, a uniform cake forms with high integrity (**e**,**f**).

A freeze-drying cycle’s primary quality indicator is the appearance of the lyophilized (lyo)-cake [57]. Depending on the integrity and shape of the cakes, samples are either accepted or rejected [57]. Collapse or meltback may indicate a loss of product or, at the very least, a decline in product quality and poor process understanding [57,58]. There are several factors that influence the lyo-cake’s appearance. On the one hand, the appearance of the lyo-cake is influenced by the characteristics of the lyoprotective agents in correlation with temperature, pressure and time. On the other hand, the possibility of vapor escaping also influences the appearance of the lyo-cake. Figure 3 discusses the different vapor escape possibilities.

Subsequent to the freezing of the samples, a vacuum is instigated within the freeze-dryer’s chamber, thereby initiating the process of sublimation [59]. During sublimation, frozen water is removed, and the water vapor must escape the SBR. Two different possibilities were investigated in our studies. On one occasion, the cover was positioned loosely on the SBR, enabling vapor to escape from the edges of the cover (Figure 3a). In the other case, shown in Figure 3b, the lid was closed to prevent vapor escaping from the edges. Instead, the membrane was perforated to allow vapor to escape through the lid. Figure 3c–f illustrate the cake appearances and the influence of the vapor escape possibility. In Figure 3c,d, the vapor during sublimation was only able to escape from the edges. Consequently, the lyo-cake exhibited morphological characteristics of collapse (Figure 3c,d; dashed circle) and meltback (Figure 3c,d; arrows), indicating that sublimation was not complete before initiating secondary drying [57]. Increasing the temperature for secondary drying to 20 °C caused the lyoprotective agent to exceed both its collapse temperature (t_c_) (collapse) and ice melting or eutectic point (meltback), resulting in a lyo-cake with visible collapse and meltback [57,60]. According to Patel et al., lyophilized samples exhibiting collapse or meltback are rejected and inappropriate for long-term storage. For aseptic reasons, the primary drying under these conditions could be extended, until all water is removed. In Figure 3e,f, vapor exit was enabled by membrane perforation directly through the lid. As seen in the images, homogenous and stable cakes were formed. This cake appearance indicates a complete sublimation cycle. High-integrity, uniform, and stable lyo-cakes, as seen in Figure 3c,d, are important for product quality, but also for facilitating the rehydration step [61]. Uniform and stable lyo-cakes comparable to Figure 3e,f are acceptable according to Patel et al. and suitable for long-term storage. To characterize the suitability for long-term storage, additional properties must be considered, such as residual water content [62].

### 2.4. Impact of Poloxamer 188 on the Rehydration

**Figure 4:**
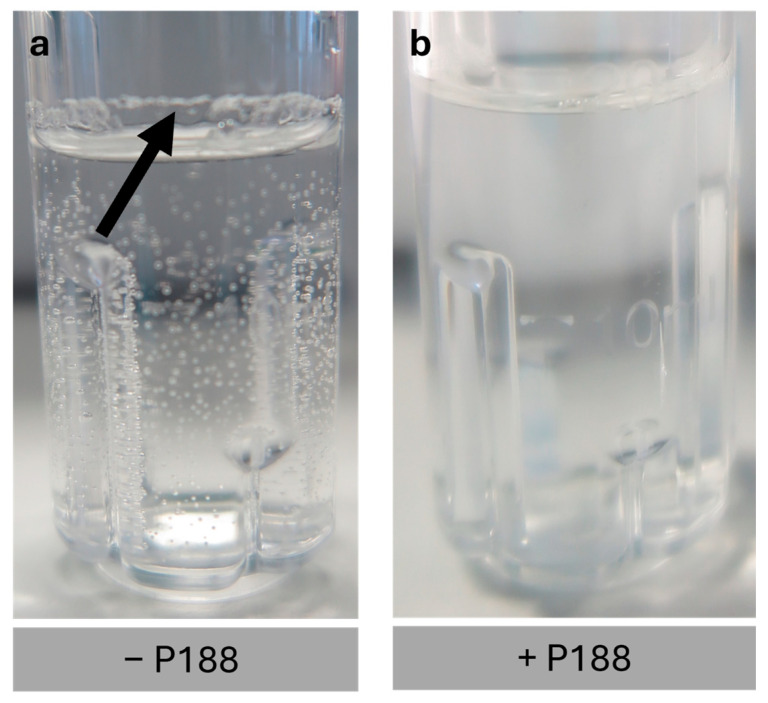
Rehydration of the cakes and impact of P188 addition on the rehydration. When P188 is not added to the formulation (**a**), bubble formation occurs. Furthermore, the UHV-alginate microcarriers stick to the inner wall of the SBR. Upon the addition of P188, bubble formation as well as the stickiness of the UHV-alginate microcarriers were reduced (**b**). Arrow in (**a**) indicates sticky UHV-alginate microcarriers.

In this section, the addition of Poloxamer 188 (P188) in the formulation was studied. The samples were rehydrated using ultrapure water. The impact of P188 is illustrated in Figure 4.

**Figure 5:**
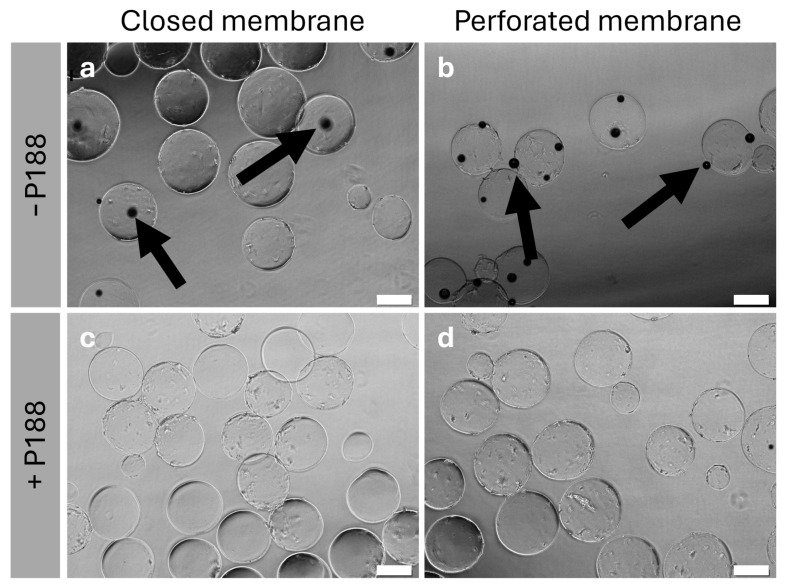
Microscopy-based analysis of the rehydrated UHV-alginate microcarriers. In absence of P188, bubbles formed (**a**,**b**). The black dots represent bubbles (arrows). Adding P188 to the formulation reduced bubble formation (**c**,**d**). Scale bar: 200 µm.

Figure 4a shows the rehydrated samples without P188 in the formulation. The absence of P188 results in significant adsorption of the protein-coated UHV-alginate microcarriers to the inner polystyrene wall of the bioreactor. Furthermore, a high number of bubbles are formed, which hampers further handling of the process since the bubbles adhere to the microcarriers (Figure 5a,b) and do not sediment. Both the bubble formation and the adsorption complicate aspiration of the lyoprotective agent. According to our findings, the addition of P188 facilitates handling. It reduces bubble formation and minimizes the adsorption of the protein-coated UHV-alginate microcarriers to the polystyrene wall of the bioreactor (Figure 4b and Figure 5c,d). This enables the aspiration of the lyoprotective agent without further steps. Figure 5c,d illustrate the reduction of bubble formation. Furthermore, the rehydrated UHV-alginate microcarriers exhibited comparable morphology and structure under all conditions.

**Figure 6:**
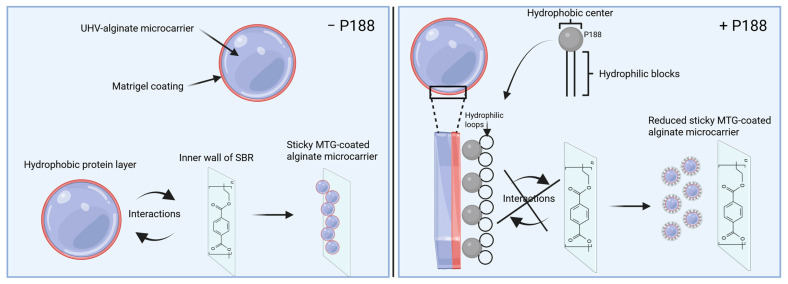
Effect of P188 on reduction of stickiness. After rehydration of the MTG-coated UHV-alginate microcarriers, the hydrophobic protein layer interacts in absence of P188 with the inner wall of the SBR, resulting in a significant amount of microcarrier being adhesive on the wall (**left side**). In the presence of P188, the hydrophobic center is interacting with the hydrophobic layer of MTG. The hydrophilic blocks of P188 form a loop, thereby sealing the hydrophobic MTG and preventing the proteins to interact with the inner wall of the SBR (**right side**). Figure created with BioRender.com.

P188 is an amphiphilic surface-active triblock copolymer that exhibits a hydrophobic core and two hydrophilic blocks linked to the core [63,64,65]. The hydrophobic core is capable of adsorbing to hydrophobic surfaces and forming loops by folding. According to Bollenbach et al., the folding creates a hydrophilic surrounding and decreases the hydrophobicity, which, in our case, leads to reduced adsorption of the protein-coated UHV-alginate microcarriers to the polystyrene wall of the bioreactor. As a surface-active detergent, P188 can migrate to interfaces, including air–water interfaces, and reduce the surface tension [66,67]. In Chang et al., surfactants decrease cell–bubble attachment for two reasons. First, it decreases the surface tension of the interface, which leads to an increase of free energy. A cell–bubble interface is formed due to this attachment. When the interface tension decreases, the amount of free energy increases, making the cell–bubble attachment less favorable. Furthermore, P188 is noted to reduce the cell’s hydrophobicity. This results in decreased hydrophobic interactions, making it less likely for bubbles to attach to the cell [67]. The mechanisms described in the literature for P188, such as the reduction of (cellular) hydrophobicity, the creation of a hydrophilic surrounding, and the mitigation of bubble formation in cell-bubble contexts, can be transferred to our findings (Figure 6).

Figure 6 illustrates the functionality of P188. In absence of P188, the hydrophobic layer of MTG interacts with the inner wall of the SBR. This leads to a binding of MTG-coated UHV-alginate microcarrier on the inner wall of SBR. In presence of P188, the hydrophobic core of P188 interacts with MTG. The hydrophilic blocks form a loop that creates a hydrophilic surrounding, thus preventing the MTG layer from interacting with the inner wall of SBR.

### 2.5. Cell Count, Cell Viability, and Cytometry Analysis

**Figure 7:**
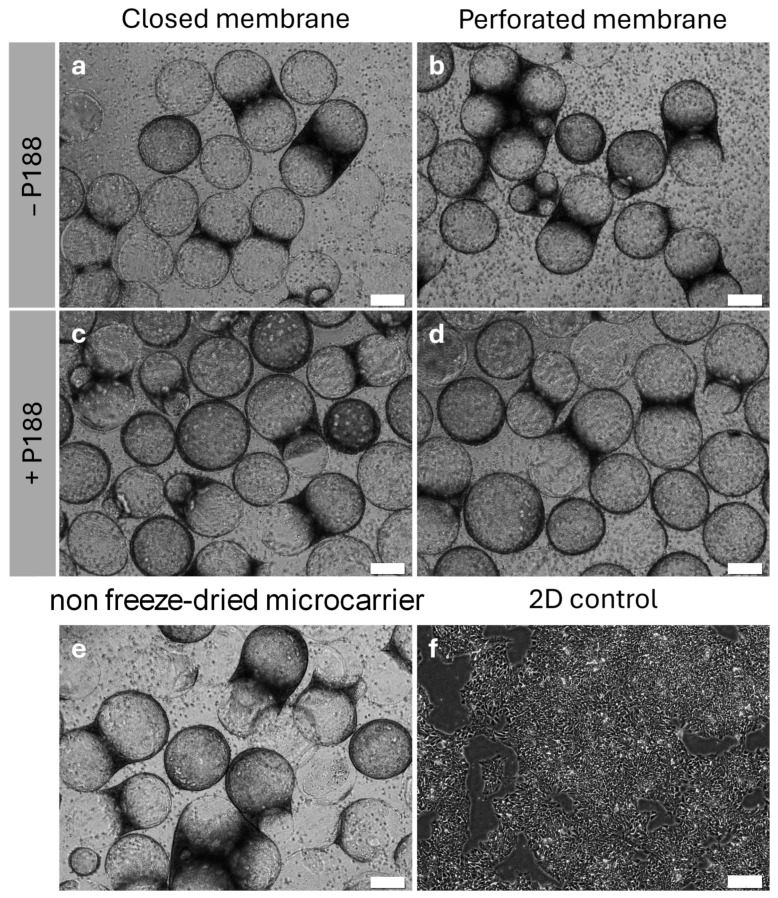
Validation of the functionality of freeze-dried and rehydrated UHV-alginate microcarriers by inoculating iPSCs and analyzing adhesion and proliferation. The images (**a**–**d**) show hiPSCs cultivated for seven days on rehydrated UHV-alginate microcarriers. (**e**) hiPSCs cultivated on non-freeze-dried UHV-alginate microcarriers. In (**f**), the iPSCs were cultivated for seven days on a control polystyrene dish. Scale bar = 200 µm.

Single cell hiPSCs were inoculated onto the rehydrated protein-coated UHV-alginate microcarriers, and the cell response—measured in terms of total cell count, cell viability, and pluripotency maintenance—was analyzed after seven days of cell expansion. Figure 7a–f show the different conditions after seven days of cell expansion.

**Figure 8:**
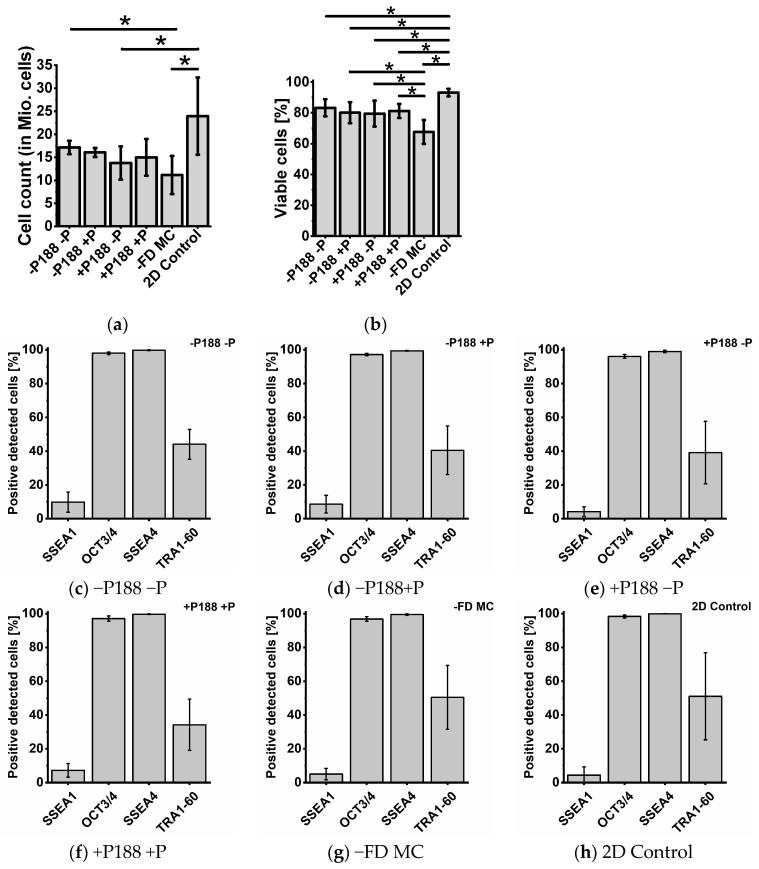
Quality control of hiPSCs after expansion on freeze-dried microcarriers. Cell count (**a**), viability (**b**), and pluripotency (**c**–**h**) of the iPSCs after seven cultivation days. Here, 2.4 × 10^6^ cells were inoculated on 40 cm^2^ UHV-alginate microcarriers and were compared to the state-of-the-art cultivation on polystyrene dishes (2D control). Flow cytometry for pluripotency analysis was performed using three pluripotency factors (OCT3/4, SSEA4, and TRA1-60) and one differentiation factor (SSEA1). Positive detected cells were determined for the following conditions: closed membrane with P188 (−P188 −P) (**c**), perforated membrane without P188 (−P188 +P) (**d**), closed membrane with P188 (+P188 −P) (**e**), perforated membrane with P188 (+P188 +P) (**f**), non-freeze-dried UHV-alginate microcarrier (−FD MC) (**g**), and the 2D control (**h**). Data are represented as means ± SDs of n = 4 (cell count and viability) and n = 3 (pluripotency) independent experiments. * indicates significant differences, *p* < 0.05.

For all conditions for the microcarrier-based expansion (Figure 7a–e), a confluency of 90% was observed. The confluency for the 2D polystyrene dishes also reached around 90% (Figure 7f). Figure 8a shows the cell count after harvesting.

A minimum of around 1.1 × 10^7^ cells was yielded from 2.4 × 10^6^ cells inoculated on the surfaces (non-freeze-dried UHV-alginate microcarrier (−FD MC)). A cell yield of around 2.4 × 10^7^ cells was harvested from 2D control. Under the freeze-dried conditions, the cell yield resulted in a minimum of around 1.4 × 10^7^ (+P188 −P) and a maximum of 1.7 × 10^7^ cells (−P188 −P). In addition, −P188 +P (1.6 × 10^7^) and +P188 +P (1.5 × 10^7^) resulted in comparable cell yields. In general, the cell yield could be increased around 5–7-fold. When comparing the fold increase after seven days of our microcarrier-based cultivation process in a SBR (5–7 fold) with the results of Rodrigues et al. using dissolvable microcarriers with Synthemax^®^ II (Corning, Inc., Corning, NY, USA) (DM-SII) coating (4-fold), it can be concluded that the overall fold increase in our SBR system is higher [68]. Additionally, considering the reported fold increase per day (FIPD) of 1.5 over a four-day cultivation period, a total fold increase of approximately 6 can be inferred from data presented by Kwok et al., which aligns well with our study [27]. Furthermore, the cell viability within the freeze-dried conditions is comparable with a range of 80–83% living cells (Figure 8b). The highest viability was observed for the 2D control at 93%, and the lowest viability was achieved for −FD MC (68% living cells). Figure 8c–h show that the flow cytometry analysis produces a similar signal for the pluripotency factors (OCT3/4, SSEA4, and TRA1-60) and the differentiation factor (SSEA1) under all conditions. The positive signal for OCT3/4 and SSEA4 is between 96% and 99.9%. The positive signal for the differentiation factor SSEA1 varies between <9.8% (−P188 −P) and <4.2% (+P188 −P), indicating a low amount of differentiated hiPSCs. The positive signal for pluripotency factor TRA1-60 is the highest for both controls 2D (51.1%) and −FD MC (50.4%). For the freeze-dried samples, it varies between 34.3% (+P188 +P) and 44.1% (−P188 −P). The cell count was increased 5–7-fold in all conditions, and the cell viability was at the minimum of 80%. Only in the −FD MC condition was a drop in cell viability observed, which might imply that the expansion processes have to be optimized for each condition. This assumption is supported by the findings of Rodrigues et al. and Kwok et al. In the study by Rodrigues et al., the total cell count peaked at approximately 3 × 10^7^ cells after five days of culture [68]. The cell count declined to around 2 × 10^7^ cells by day seven [68]. This suggests that the microcarrier had already reached full confluence by day five and that cells should have been harvested at that point. Similarly, Kwok et al. demonstrated that, for single cell cultures on UHV-alginate microcarriers, full confluence was achieved after only four days of cultivation [27]. Since the −FD MC condition is comparable to Kwok et al., a reduction in the cultivation period or alternatively an increase in growth surface provided by microcarriers might increase total cell count and viability by preventing over-confluence. Nevertheless, each condition within these microcarrier-based cultivation processes generates cell numbers sufficient for subsequent applications, such as toxicity screening or compound testing [69,70]. A comprehensive overview of the key results from the cell expansion investigations is provided in Table 2.

According to the flow cytometry analysis, the hiPSCs were able to maintain their pluripotency status. When compared to the literature values reported by Kwok et al., where hiPSCs were cultivated on UHV-alginate microcarriers, the positive detected signals of pluripotency factor SSEA4 were found to be comparable (>99%). In the case of OCT3/4, the proportion of positively detected cells in our findings was slightly higher, exceeding 96%. In our results, positive detected cells for TRA1-60 was significantly lower, by approximately 50%, compared to the values reported by Kwok et al. [27]. The low positive signal for the pluripotency factor TRA1-60 might be an artefact. During fixation using formaldehyde and storage for more than four weeks, shrinkage of the cells occurs, which leads to an inaccessibility for the antibodies to attach to the pluripotency factor. Furthermore, during double staining, the path to the antigen might be blocked. The proportion of SSEA1-positive detected cells in our findings (4.38–9.81%) was comparably low to the values reported by Kwok et al. (<5%) [27]. According to the pluripotency and differentiation signals, the hiPSCs could maintain their pluripotency status. Nevertheless, to confirm their pluripotency status differentiation, trilineage differentiation processes have to be performed. In total, these findings are promising, and the next steps for implementing a “ready-to-use” bioreactor-integrated functional freeze-drying process for the expansion of iPSCs can be initiated.

## 3. Conclusions

In this study, the first investigations toward a “ready-to-use” bioreactor-integrated functional freeze-drying process for protein-coated UHV-alginate microcarriers were performed. Initially, a simple simulation was conducted, demonstrating the shorter primary drying duration of the 22° oriented SBR setup compared to the 90° oriented SBR configuration. However, due to its simplified assumptions, this model cannot predict absolute drying times or optimize process parameters. The freeze-drying process was carried out directly in the SBR. This reduced transferring steps, reducing functional surface loss. The addition of P188 had no negative impact on the morphology or the functionality of the protein-coated UHV-alginate microcarriers. P188 reduced bubble formation and protein adsorption, which facilitated the process. The sublimation time for the condition with an intact membrane was not enough to complete water removal before switching to the secondary drying. Due to aseptic reasons, the time could be extended, or other possibilities must be considered to ensure sterility. Another important aspect for further investigations is the possibility of completing the freeze-drying process and closing the SBR before removing them from the chamber to enable long-term storage under vacuum conditions.

## 4. Materials and Methods

### 4.1. In Silico Verification of SBR Orientation During Drying Process

To verify the faster drying rate of the 22° orientation (“ready-to-use”) SBR setup (see Figure 1b), a simple sublimation simulation was established using the FEM software COMSOL Multiphysics^®^ v6.2 (COMSOL AB, Stockholm, Sweden) on a workstation containing a 16-core 11th Gen Intel^®^ Core™ i9-11900K CPU and 64 GB of RAM. The primary drying processes in the upright (90° orientation) and tilted (22° orientation) SBR setups were compared. The change in normalized remaining ice content is modeled as follows:

(1) $$\frac{\delta s}{\delta t}=\frac{-\dot{m}}{\rho_{ice}\epsilon_{0}},$$

where $\epsilon_{0}$ is the initial porosity (dimensionless, representing the fraction of volume occupied by ice), and $\rho_{ice}$ is the density of ice, and $\dot{m}$ is the sublimated mass flux per unit volume and time. It is approximated using the Hertz-Knudsen equation, neglecting secondary mass transfer effects, as follows:

(2) $$\dot{m}=S_{v}\alpha \left(p_{v}-p\right)\sqrt{\frac{M_{v}}{2\pi RT}}.$$

Here, *T* is temperature, $M_{v}$ is the molar mass of vapor, $S_{v}$ is the specific surface area, *R* is the universal gas constant, α is an evaporation coefficient, *p* is the ambient pressure, and $p_{v}$ is the vapor pressure. It is calculated using an empirical equation (valid for *T* > 110 K) from Murphy and Koop as follows [71]:

(3) $$p_{v}=\exp\left(9.550426-\frac{5723.265}{T}+3.53068\ln\left(T\right)-0.00728332T\right).$$

The loss in heat due to sublimation is described as follows:

(4) $${\dot{Q}}_{subl}=\dot{m}\Delta H_{s},$$

where $\Delta H_{s}$ is the heat of sublimation.

The overall vapor flow and heat transfer were modeled using the “Darcy’s Law” and “Heat Transfer in Solids and Fluids” modules in COMSOL. SBR geometry and solution height were based on laboratory measurements. Drying duration, ambient temperature, and pressure are identical to the experimental setup given in Table 1 for primary drying. For simplicity, remaining necessary parameters (e.g., heat capacity, thermal conductivity, density, etc.) were assumed to match pure ice. This assumption is justified, as the model aims to compare drying rates between vial geometries rather than to precisely replicate experimental conditions.

### 4.2. Cell Culture

The cell line used for the expansion processes was UKBi005-A (BioSample ID: SAMEA4584351, listed on hpscreg.eu), which is an hiPSC cell line registered with the European Bank for Induced Pluripotent Stem Cells (EBiSC). The cells were maintained and cultivated on MTG-coated (Corning, CA, USA) 6 cm cell culture dishes (Nunclon^TM^ Delta, Thermo Fisher Scientific, Waltham, MA, USA or Greiner Bio One GmbH, Frickenhausen, Germany) using mTeSR^TM^1 medium (Stemcell Technologies, Vancouver, BC, Canada). The medium was changed every day, and the cell quality was monitored microscopically. At a confluency of around 80–90%, the cells were dissociated with 0.5 mM ethylenediaminetetraacetic acid (EDTA, Invitrogen, Waltham, MA, USA) for maintenance. For the single cell expansion processes, the cells were cultivated on 15 cm cell culture dishes (Corning, USA) and dissociated according to Section 4.5.

### 4.3. Hydrogel: UHV-Alginate Microcarrier

One batch of MTG-coated UHV-alginate microcarrier (Alginatec, Riedenheim, Germany) was used for the freeze-drying and expansion processes. The alginates were extracted from brown algae *Lessonia nigrescens* (LN) and *Lessonia trabeculata* (LT) and modified according to Gepp et al. [31]. A 1:1 (*v*/*v*) mixture of the batches LN62 (0.65% solution, solved in 0.9% NaCl) and LT26 (0.65% solution, solved in 0.9% NaCl) were used to produce the microcarrier. The diameter of the microcarrier was about 356–370 µm. Here, 40 cm^2^ cultivation surface was used per condition.

### 4.4. Freeze Drying and Rehydration

Briefly, 40 cm^2^ MTG-coated UHV-alginate microcarriers were transferred together with 100 mg/mL trehalose (trehalose dihydrate, EMPROVE^®^ EXPERT, Ph. Eur., Sigma Merck, Darmstadt, Germany) (diluted in 0.9% NaCl (B. Braun, Melsungen, Germany)) into the SBR. For the samples without 10% P188 (Sigma Merck), the total volume was 16 mL. When P188 was added, the total volume was 17.6 mL, resulting in a final P188 concentration of 0.9%. The P188 concentration of nearly 1% was selected because it has already been successfully used in the context of cell cryopreservation [72,73]. Furthermore, the vessels were positioned inside the freeze-dryer (Alpha 1-4 LSC_plus_, Martin Christ GmbH, Osterode am Harz, Germany) (see Figure 1b). A trial freeze-drying cycle was conducted by Biopharma Process Systems (Winchester, UK) and further modified (see Table 3). The samples were stored in the freeze-dryer at 20 °C and 0.1 mbar until cell availability. After freeze-drying, the samples were rehydrated according to the initial volume (16.0 mL or 17.6 mL) with ultrapure water (Milli-Q^®^, Merck Millipore, Darmstadt, Germany). Samples for microcarrier morphology and bubble formation analysis were taken afterwards, and the samples were incubated overnight at 37 °C and 5% CO_2_. The incubation step was followed by centrifugation until the microcarriers were sedimented. Subsequently, the microcarriers were washed 1–2 times with DMEM/F12 −/− (Gibco^TM^, Thermo Fisher Scientific, Waltham, MA, USA) before adding single cells for their expansion.

### 4.5. Dissociation of Single Cells

The hiPSCs were dissociated from the cell culture dishes using TrypLE^TM^ (Gibco, Thermo Fisher Scientific, Waltham, MA, USA). First, the cells were washed twice with DMEM −/− or DPBS −/− (Gibco^TM^, Thermo Fisher Scientific, Waltham, MA, USA) followed by a 5 min TrypLE^TM^ Select (Gibco, Thermo Fisher Scientific, Waltham, MA, USA) dissociation step inside the incubator. The process was stopped by adding mTeSR^TM^1 to the cell culture dish. After detachment, the cells were centrifuged (3 min, 250× *g*), and the supernatant was discarded. The cell pellet was resuspended with mTeSR^TM^1 supplied with 10 µM ROCK inhibitor Y-27632 to prevent apoptosis of the single cells.

### 4.6. Cultivation of hiPSCs in a Suspension Bioreactor

The hiPSCs were cultivated in SBRs (CEROtube vessels, OLS, Bremen, Germany) in a bioreactor system CERO 3D (OLS, Bremen, Germany) at 37 °C and 5% CO_2_. Starting with the inoculation, 60,000 cells per cm^2^ were inoculated on the microcarrier. The inoculation volume was around 4.3 mL. The detailed program is listed in Table 4 and was previously used in Kwok et al. [27] The volume was filled up to 10 mL after one day, and 50% of the medium was changed every day. Over the weekend, the volume was filled up to 20 mL. The cells were expanded for seven days. To ensure a seven-day expansion process for the cell culture dish control, the cells were split on day 2 or 3 from one dish to two dishes and split again at day 5 into four dishes. The dissociation was performed using EDTA. The cultivation medium included Pen/Strep (1:100, Fisher Scientific, Waltham, MA, USA). For harvesting, the cells were dissociated with improved EDTA (iEDTA, developed at Fraunhofer IBMT). Before adding the dissociation agent, the cells were washed twice with DPBS −/−. The harvesting step was initiated inside the CERO system for 20 min. When the cells were detached, they were separated from the microcarrier using a 200 µm strainer (pluriSelect Life Science, Leipzig, Germany) followed by a cell count and viability analysis using NucleoCounter^®^ NC-200^TM^ system (ChemoMetec, Lillerød, Denmark). Subsequently, the cells were washed twice with PBS −/− and fixed for 15–20 min with 4% formaldehyde (Cytofix^TM^, BD, Franklin Lakes, NJ, USA). After fixation, the cells were washed once with staining buffer (BD, Franklin Lakes, NJ, USA) and stored in staining buffer at 4 °C until further usage.

### 4.7. Flow Cytometry Analysis

The Human and Mouse Pluripotent Stem Cell Analysis Kit (BD, Franklin Lakes, NJ, USA) was used for flow cytometry analysis. Additionally, TRA1-60 (see Table 5) was used as a further pluripotency factor. First, the fixed cells were washed in 5 mL PBS −/−, followed by a washing step performed two times in 1 mL Perm/Wash buffer, which was then resuspended in 0.5 mL FACS buffer. Afterwards, the cells were stained with 20 µL of antibodies for SSEA4, OCT3/4, and SSEA1 and 5 µL for TRA1-60 per 1 × 10^6^ cells (see Table 5). The stained cells were incubated for 30 min at 4 °C. After incubation, 4 mL FACS buffer was added to the samples, and the samples were centrifuged for 3 min at 250× *g*. Last, the samples were resuspended in 0.3 mL FACS buffer. Flow cytometry analysis was performed using a BD FACS Canto^TM^ II flow cytometer (BD Bioscience, Franklin Lakes, NJ, USA).

### 4.8. Statistical Evaluation

The statistical evaluation was performed using OriginPro (Version 2021, OriginLab Corporation, Northampton, MA, USA). For total cell count data, normality was partially rejected; therefore, a Kruskal-Wallis test followed by Dunn’s post hoc test was used to asses statistical significance (*p* < 0.05). In contrast, the viability data showed no significant deviation from normal distribution. Accordingly, a one-way ANOVA followed by Tukey’s post hoc test was performed, also with a significance level of *p* < 0.05.

## Figures and Tables

**Table 1:** Overview of commercially available cultivation surfaces with different dimensions, materials, and stiffnesses. Although the table highlights representative examples, it should not be considered a comprehensive listing of all “ready-to-use” cell culture surfaces presently available commercially.

Brand Name	Manufacturer or Reference	Dimension	Type	Material	Stiffness
Nunc^TM^	Thermo Fisher Scientific Inc. (Waltham, MA, USA) [8,9]	Flat	Well plates, culture dishes, flasks	Polystyrene (Nunclon delta)	2.28–3.28 GPa [10]
Elastically Supported Surface (ESS)	ibidi GmbH (Gräfelfing, Germany) [11,12]	Flat	Culture dishes	Polydimethylsiloxane (PDMS) with glass bottom	1.5, 15. 28 kPa
Softwell^TM^, Petrisoft, Softslip, Soft Flask	Matrigen, LLC (Irvine, CA, USA) [13]	flat	Well plates, culture dishes, flasks	Polyacrylamide-based gel bound on polystyrene or glass	0.1–100 kPa
CytoSoft^®^	Advanced BIOMATRIX, Inc. (San Diego, CA, USA) [14]	Flat	Well plates, flasks	PDMS (with glass bottom)	0.2–64 kPa
MecaChips^®^	Cell&Soft, SAS (Grenoble, France) [15]	Flat	Culture dishes, well plates	ECM Proteins or synthetic amino acid	1–25 kPa
Cytodex^TM^ 1	Cytiva (Marlborough, MA, USA) [16,17]	Sphere	Microcarrier	Diethylaminomethyl (DEAE)-groups coupled on dextran	n.d.
Cytodex^TM^ 3	Cytiva (Marlborough, MA, USA) [17,18]	Sphere	Microcarrier	Denatured collagen coated on dextran	n.d.
Cytopore^TM^ 1	Cytiva (Marlborough, MA, USA) [17,19]	Sphere	Microcarrier	DEAE groups coupled on cellulose	n.d.
Solohill^®^	Sartorius AG (Göttingen, Germany) [20,21]	Sphere	Microcarrier	(Modified or cross-linked) polystyrene and available with (surface modified) type 1 porcine collagen coating	n.d.
Corning^®^ Microcarrier	Corning, Inc. (Corning, NY, USA) [22]	Sphere	Microcarrier	Polystyrene coated with, e.g., Synthemax II^®^	n.d.
Dissolvable Microcarriers	IamFluidics B.V. (Enschede, The Netherlands) and Rousselot Biomedical B.V. (Son en Breugel, The Netherlands) [23]	Sphere	Dissolvable microcarrier	Sodium alginate coated with denatured collagen	n.d.
CultiSpher^®^: G, GL, S	Percell Biolytica, AB (Åstorp, Sweden) [24,25,26]	Sphere	Microcarrier	Gelatin	n.d.

**Table 2:** Summary of the key findings of the cell expansion studies, including cell count, viability, and pluripotency. Data are represented as means ± SDs of n = 4 (cell count and viability) and n = 3 (pluripotency) independent experiments.

Condition	Cell Count [×10^7^]	Viability [%]	Pluripotency or Differentiation Factor (Positive Detected Cells [%])			
			OCT3/4	SSEA4	TRA1-60	SSEA1
−P188 −P	1.71 ± 0.14	83.26 ± 5.54	97.95 ± 0.79	99.73 ± 0.29	44.1 ± 8.85	9.81 ± 5.94
−P188 +P	1.6 ± 0.1	80.09 ± 6.8	97.2 ± 0.65	99.37 ± 0.23	40.49 ± 14.41	8.63 ± 5.23
+P188 −P	1.38 ± 0.36	79.51 ± 8.39	96.1 ± 1.11	99 ± 0.72	39.16 ± 18.52	4.17 ± 2.86
+P188 +P	1.5 ± 0.4	81.19 ± 4.58	97.03 ± 1.55	99.67 ± 0.23	34.25 ± 15.23	7.23 ± 4.05
−FD MC	1.11 ± 0.41	67.62 ± 7.71	96.76 ± 1.39	99.43 ± 0.47	50.44 ± 18.96	5.03 ± 3.37
2D control	2.39 ± 0.84	93.16 ± 2.42	98.31 ± 0.86	99.93 ± 0.06	51.11 ± 25.8	4.38 ± 4.95

**Table 3:** Process parameters for the freeze-drying cycles of UHV-alginate microcarriers.

Parameter	Ramp	Hold	Ramp	Hold	Ramp	Hold
Temperature [°C]	Maximum	Maximum	Maximum	Maximum	20	20
Pressure [mbar]	/	/	0.05	0.05	0.1	0.1
Time [h]	2.5	12	1.5	30	5	≥8
Event	Freezing	Freezing	Primary drying	Primary drying	Secondary drying	Secondary drying

**Table 4:** Program for dynamic expansion processes of single-cell hiPSCs in CERO SBRs. The program was previously used in Kwok et al. [27].

Program	Rotation Period [s]	Rotation Speed [rpm]	Agitation Period [min]	Agitation Pause [min]	Duration
Inoculation	4	40	2	5	12 h
Cultivation	4	40	2	/	7 days
Harvesting	5	60	/	/	20 min

**Table 5:** Pluripotency and differentiation antibodies and isotype controls used for flow cytometry analysis.

Antibodies	Dilution
Alexa Fluor^®^ 647 Rat anti-SSEA4 Alexa 647 Mouse IgG3, _K_ Isotype Control	20 µL per million cells
PerCP-Cy^TM^ 5.5 Mouse anti-OCT3/4 PerCP-Cy^TM^ 5.5 Mouse IgG1, _K_ Isotype Control	20 µL per million cells
PE Rat anti-SSEA1 PE Mouse IgM, _K_ Isotype Control	20 µL per million cells
Alexa Fluor^®^ 488 anti-human TRA1-60 Alexa 488 Mouse IgM, _K_ Isotype Control	5 µL per million cells

## Data Availability

The original contributions presented in the study are included in the article; further inquiries can be directed to the corresponding author.

## References

[1] Schulz A., Gepp M.M., Stracke F., von Briesen H., Neubauer J.C., Zimmermann H. (2019). Tyramine-conjugated alginate hydrogels as a platform for bioactive scaffolds. J. Biomed. Mater. Res. A.

[2] Kapałczyńska M., Kolenda T., Przybyła W., Zajączkowska M., Teresiak A., Filas V., Ibbs M., Bliźniak R., Łuczewski Ł., Lamperska K. (2018). 2D and 3D cell cultures—A comparison of different types of cancer cell cultures. Arch. Med. Sci..

[3] Pampaloni F., Reynaud E.G., Stelzer E.H.K. (2007). The third dimension bridges the gap between cell culture and live tissue. Nat. Rev. Mol. Cell Biol..

[4] Jensen C., Teng Y. (2020). Is It Time to Start Transitioning From 2D to 3D Cell Culture?. AAPS PharmSciTech.

[5] Bellin M., Marchetto M.C., Gage F.H., Mummery C.L. (2012). Induced pluripotent stem cells: The new patient?. Nat. Rev. Mol. Cell Biol..

[6] Vallabhaneni H., Shah T., Shah P., Hursh D.A. (2023). Suspension culture on microcarriers and as aggregates enables expansion and differentiation of pluripotent stem cells (PSCs). Cytotherapy.

[7] Badenes S.M., Fernandes T.G., Rodrigues C.A.V., Diogo M.M., Cabral J.M.S. (2016). Microcarrier-based platforms for in vitro expansion and differentiation of human pluripotent stem cells in bioreactor culture systems. J. Biotechnol..

[8] Steele J.G., Dalton B.A., Johnson G., Underwood P.A. (1993). Polystyrene chemistry affects vitronectin activity: An explanation for cell attachment to tissue culture polystyrene but not to unmodified polystyrene. J. Biomed. Mater. Res..

[9] Zeiger A.S., Hinton B., van Vliet K.J. (2013). Why the dish makes a difference: Quantitative comparison of polystyrene culture surfaces. Acta Biomater..

[10] Acevedo-Acevedo S., Crone W.C. (2015). Substrate stiffness effect and chromosome missegregation in hIPS cells. J. Negat. Results Biomed..

[11] Strale P.-O., Azioune A., Bugnicourt G., Lecomte Y., Chahid M., Studer V. (2016). Multiprotein Printing by Light-Induced Molecular Adsorption. Adv. Mater..

[12] Ho K.K.Y., Buschhaus J.M., Zhang A., Cutter A.C., Humphries B., Luker G.D. (2025). Substrate stiffness regulates triple-negative breast cancer signaling through CXCR4 receptor dynamics. bioRxiv.

[13] Matrigen. https://store.matrigen.com/.

[14] Advanced BioMatrix—CytoSoft^®^ Rigidity Plates. https://advancedbiomatrix.com/cytosoft/.

[15] Visonà A., Cavalaglio S., Labau S., Soulan S., Joisten H., Berger F., Dieny B., Morel R., Nicolas A. (2024). Substrate softness increases magnetic microdiscs-induced cytotoxicity. Nanoscale Adv..

[16] van Wezel A.L. (1967). Growth of cell-strains and primary cells on micro-carriers in homogeneous culture. Nature.

[17] Chen X.-Y., Chen J.-Y., Tong X.-M., Mei J.-G., Chen Y.-F., Mou X.-Z. (2020). Recent advances in the use of microcarriers for cell cultures and their ex vivo and in vivo applications. Biotechnol. Lett..

[18] Sart S., Agathos S.N., Li Y. (2013). Engineering stem cell fate with biochemical and biomechanical properties of microcarriers. Biotechnol. Prog..

[19] Spearman M., Rodriguez J., Huzel N., Butler M. (2005). Production and glycosylation of recombinant beta-interferon in suspension and cytopore microcarrier cultures of CHO cells. Biotechnol. Prog..

[20] Li B., Wang X., Wang Y., Gou W., Yuan X., Peng J., Guo Q., Lu S. (2015). Past, present, and future of microcarrier-based tissue engineering. J. Orthop. Translat..

[21] Tavassoli H., Alhosseini S.N., Tay A., Chan P.P.Y., Weng Oh S.K., Warkiani M.E. (2018). Large-scale production of stem cells utilizing microcarriers: A biomaterials engineering perspective from academic research to commercialized products. Biomaterials.

[22] Rafiq Q.A., Coopman K., Nienow A.W., Hewitt C.J. (2016). Systematic microcarrier screening and agitated culture conditions improves human mesenchymal stem cell yield in bioreactors. Biotechnol. J..

[23] IamFluidics Dissolvable Microcarriers|IamFluidics. https://iamfluidics.com/products/dissolvable-microcarriers/.

[24] Simão V.A., Brand H., Da Silveira-Antunes R.N., Fukasawa J.T., Leme J., Tonso A., Ribeiro-Paes J.T. (2023). Adipose-derived stem cells (ASCs) culture in spinner flask: Improving the parameters of culture in a microcarrier-based system. Biotechnol. Lett..

[25] Yuan Y., Kallos M.S., Hunter C., Sen A. (2014). Improved expansion of human bone marrow-derived mesenchymal stem cells in microcarrier-based suspension culture. J. Tissue Eng. Regen. Med..

[26] Larsson A.P., Briheim K., Hanna V., Gustafsson K., Starkenberg A., Vintertun H.N., Kratz G., Junker J.P.E. (2021). Transplantation of autologous cells and porous gelatin microcarriers to promote wound healing. Burns.

[27] Kwok C.K., Sébastien I., Hariharan K., Meiser I., Wihan J., Altmaier S., Karnatz I., Bauer D., Fischer B., Feile A. (2022). Scalable expansion of iPSC and their derivatives across multiple lineages. Reprod. Toxicol..

[28] Storz H., Müller K.J., Ehrhart F., Gómez I., Shirley S.G., Gessner P., Zimmermann G., Weyand E., Sukhorukov V.L., Forst T. (2009). Physicochemical features of ultra-high viscosity alginates. Carbohydr. Res..

[29] Storz H., Zimmermann U., Zimmermann H., Kulicke W.-M. (2010). Viscoelastic properties of ultra-high viscosity alginates. Rheol. Acta.

[30] Zimmermann H., Shirley S.G., Zimmermann U. (2007). Alginate-based encapsulation of cells: Past, present, and future. Curr. Diab. Rep..

[31] Gepp M.M., Fischer B., Schulz A., Dobringer J., Gentile L., Vásquez J.A., Neubauer J.C., Zimmermann H. (2017). Bioactive surfaces from seaweed-derived alginates for the cultivation of human stem cells. J. Appl. Phycol..

[32] Dodero A., Pianella L., Vicini S., Alloisio M., Ottonelli M., Castellano M. (2019). Alginate-based hydrogels prepared via ionic gelation: An experimental design approach to predict the crosslinking degree. Eur. Polym. J..

[33] Saitoh S., Araki Y., Kon R., Katsura H., Taira M. (2000). Swelling/deswelling mechanism of calcium alginate gel in aqueous solutions. Dent. Mater. J..

[34] Boontheekul T., Kong H.-J., Mooney D.J. (2005). Controlling alginate gel degradation utilizing partial oxidation and bimodal molecular weight distribution. Biomaterials.

[35] Chang L.L., Pikal M.J. (2009). Mechanisms of protein stabilization in the solid state. J. Pharm. Sci..

[36] Mensink M.A., Frijlink H.W., van der Voort Maarschalk K., Hinrichs W.L.J. (2017). How sugars protect proteins in the solid state and during drying (review): Mechanisms of stabilization in relation to stress conditions. Eur. J. Pharm. Biopharm..

[37] Pardeshi S.R., Deshmukh N.S., Telange D.R., Nangare S.N., Sonar Y.Y., Lakade S.H., Harde M.T., Pardeshi C.V., Gholap A., Deshmukh P.K. (2023). Process development and quality attributes for the freeze-drying process in pharmaceuticals, biopharmaceuticals and nanomedicine delivery: A state-of-the-art review. Future J. Pharm. Sci..

[38] Soofi S.S., Last J.A., Liliensiek S.J., Nealey P.F., Murphy C.J. (2009). The elastic modulus of Matrigel as determined by atomic force microscopy. J. Struct. Biol..

[39] Aisenbrey E.A., Murphy W.L. (2020). Synthetic alternatives to Matrigel. Nat. Rev. Mater..

[40] Talbot N.C., Caperna T.J. (2015). Proteome array identification of bioactive soluble proteins/peptides in Matrigel: Relevance to stem cell responses. Cytotechnology.

[41] Diebels S., Gepp M.M., Meiser I., Roland M., Stracke F., Zimmermann H. (2021). A multiphase model for the cross-linking of ultra-high viscous alginate hydrogels. Proc. Appl. Math. Mech..

[42] Fu S., Buckner I.S., Block L.H. (2014). Inter-grade and inter-batch variability of sodium alginate used in alginate-based matrix tablets. AAPS PharmSciTech.

[43] Smaniotto F., Prosapio V., Zafeiri I., Spyropoulos F. (2020). Freeze drying and rehydration of alginate fluid gels. Food Hydrocoll..

[44] Merivaara A., Zini J., Koivunotko E., Valkonen S., Korhonen O., Fernandes F.M., Yliperttula M. (2021). Preservation of biomaterials and cells by freeze-drying: Change of paradigm. J. Control. Release.

[45] Odziomek K., Drabczyk A.K., Kościelniak P., Konieczny P., Barczewski M., Bialik-Wąs K. (2024). The Role of Freeze-Drying as a Multifunctional Process in Improving the Properties of Hydrogels for Medical Use. Pharmaceuticals.

[46] Pansare S.K., Patel S.M. (2019). Lyophilization Process Design and Development: A Single-Step Drying Approach. J. Pharm. Sci..

[47] Grenier J., Duval H., Barou F., Lv P., David B., Letourneur D. (2019). Mechanisms of pore formation in hydrogel scaffolds textured by freeze-drying. Acta Biomater..

[48] Grenier J., Duval H., Lv P., Barou F., Le Guilcher C., Aid R., David B., Letourneur D. (2022). Interplay between crosslinking and ice nucleation controls the porous structure of freeze-dried hydrogel scaffolds. Biomater. Adv..

[49] Zhang X., Wang X., Fan W., Liu Y., Wang Q., Weng L. (2022). Fabrication, Property and Application of Calcium Alginate Fiber: A Review. Polymers.

[50] Shapiro L., Cohen S. (1997). Novel alginate sponges for cell culture and transplantation. Biomaterials.

[51] Tal Y., van Rijn J., Nussinovitch A. (1997). Improvement of Structural and Mechanical Properties of Denitrifying Alginate Beads by Freeze-Drying. Biotechnol. Prog..

[52] Łabowska M.B., Skrodzka M., Sicińska H., Michalak I., Detyna J. (2023). Influence of Cross-Linking Conditions on Drying Kinetics of Alginate Hydrogel. Gels.

[53] Chiu T.-H., Wu S.-Y., Yang Y.-C., Yan C.-J., Yeh Y.-C. (2024). Fabrication of Luminescent Triple-Cross-Linked Gelatin/Alginate Hydrogels through Freezing-Drying-Swelling and Freezing-Thawing Processes. Biomacromolecules.

[54] Zohar-Perez C., Chet I., Nussinovitch A. (2004). Irregular textural features of dried alginate–filler beads. Food Hydrocoll..

[55] Santana B.P., Nedel F., Perelló Ferrúa C., Marques e Silva R., Da Silva A.F., Demarco F.F., Lenin Villarreal Carreño N. (2015). Comparing different methods to fix and to dehydrate cells on alginate hydrogel scaffolds using scanning electron microscopy. Microsc. Res. Tech..

[56] Chui C.-Y., Bonilla-Brunner A., Seifert J., Contera S., Ye H. (2019). Atomic force microscopy-indentation demonstrates that alginate beads are mechanically stable under cell culture conditions. J. Mech. Behav. Biomed. Mater..

[57] Patel S.M., Nail S.L., Pikal M.J., Geidobler R., Winter G., Hawe A., Davagnino J., Rambhatla Gupta S. (2017). Lyophilized Drug Product Cake Appearance: What Is Acceptable?. J. Pharm. Sci..

[58] Lu X., Kulkarni S.S., Dong H., Tang Y., Yi L., Gupta S. (2023). Freezing process influences cake appearance of a lyophilized amorphous protein formulation with low solid content and high fill configuration. Int. J. Pharm..

[59] Tchessalov S., Maglio V., Kazarin P., Alexeenko A., Bhatnagar B., Sahni E., Shalaev E. (2023). Practical Advice on Scientific Design of Freeze-Drying Process: 2023 Update. Pharm. Res..

[60] Ohori R., Yamashita C. (2017). Effects of temperature ramp rate during the primary drying process on the properties of amorphous-based lyophilized cake, Part 1: Cake characterization, collapse temperature and drying behavior. J. Drug Deliv. Sci. Technol..

[61] Haeuser C., Goldbach P., Huwyler J., Friess W., Allmendinger A. (2018). Imaging Techniques to Characterize Cake Appearance of Freeze-Dried Products. J. Pharm. Sci..

[62] Duralliu A., Matejtschuk P., Williams D.R. (2018). Humidity induced collapse in freeze dried cakes: A direct visualization study using DVS. Eur. J. Pharm. Biopharm..

[63] Torcello-Gómez A., Wulff-Pérez M., Gálvez-Ruiz M.J., Martín-Rodríguez A., Cabrerizo-Vílchez M., Maldonado-Valderrama J. (2014). Block copolymers at interfaces: Interactions with physiological media. Adv. Colloid Interface Sci..

[64] Bollenbach L., Buske J., Mäder K., Garidel P. (2022). Poloxamer 188 as surfactant in biological formulations—An alternative for polysorbate 20/80?. Int. J. Pharm..

[65] Yakaew S., Luangpradikun K., Phimnuan P., Nuengchamnong N., Kamonsutthipaijit N., Rugmai S., Nakyai W., Ross S., Ungsurungsei M., Viyoch J. (2022). Investigation into poloxamer 188-based cubosomes as a polymeric carrier for poor water-soluble actives. J. Appl. Polym. Sci..

[66] Peng H., Ali A., Lanan M., Hughes E., Wiltberger K., Guan B., Prajapati S., Hu W. (2016). Mechanism investigation for poloxamer 188 raw material variation in cell culture. Biotechnol. Prog..

[67] Chang D., Fox R., Hicks E., Ferguson R., Chang K., Osborne D., Hu W., Velev O.D. (2017). Investigation of interfacial properties of pure and mixed poloxamers for surfactant-mediated shear protection of mammalian cells. Colloids Surf. B Biointerfaces.

[68] Rodrigues A.L., Rodrigues C.A.V., Gomes A.R., Vieira S.F., Badenes S.M., Diogo M.M., Cabral J.M.S. (2019). Dissolvable Microcarriers Allow Scalable Expansion and Harvesting of Human Induced Pluripotent Stem Cells Under Xeno-Free Conditions. Biotechnol. J..

[69] Burnett S.D., Blanchette A.D., Grimm F.A., House J.S., Reif D.M., Wright F.A., Chiu W.A., Rusyn I. (2019). Population-based toxicity screening in human induced pluripotent stem cell-derived cardiomyocytes. Toxicol. Appl. Pharmacol..

[70] MacMullen C., Davis R.L. (2021). High-Throughput Phenotypic Assay for Compounds That Influence Mitochondrial Health Using iPSC-Derived Human Neurons. SLAS Discov..

[71] Murphy D.M., Koop T. (2005). Review of the vapour pressures of ice and supercooled water for atmospheric applications. Q. J. R. Meteorol. Soc..

[72] Mehdipour M., Daghigh Kia H., Martínez-Pastor F. (2020). Poloxamer 188 exerts a cryoprotective effect on rooster sperm and allows decreasing glycerol concentration in the freezing extender. Poult. Sci..

[73] Schulz J.C., Germann A., Kemp-Kamke B., Mazzotta A., von Briesen H., Zimmermann H. (2012). Towards a xeno-free and fully chemically defined cryopreservation medium for maintaining viability, recovery, and antigen-specific functionality of PBMC during long-term storage. J. Immunol. Methods.

